# Autochthonous cutaneous sparganosis in Brazil: report of two cases^☆^

**DOI:** 10.1016/j.abd.2026.501430

**Published:** 2026-08-12

**Authors:** Ana Carolina Cortes Ferreira, Mariana Bezerra Benevides Brandão, Emyle Vitoria Pereira Sousa, Mariana Serdeira Arbex Pinto, Kátia Maria Serdeira Arbex, Thiago Jeunon de Sousa Vargas

**Affiliations:** a Service of Dermatology, Universidade de Gurupi, Gurupi, TO, Brazil; b Service of Internal Medicine, Hospital do Servidor Público Estadual, Instituto de Assistência Médica ao Servidor Público Estadual, São Paulo, SP, Brazil; c Department of Gastroenterology, Clínica Gastrovale, Volta Redonda, RJ, Brazil; d Service of Dermatopathology, ID - Investigação em Dermatologia, Rio de Janeiro, RJ, Brazil

Dear Editor,

Sparganosis is a rare zoonosis caused by the plerocercoid larva of cestodes of the genus *Spirometra*.^1^ Adult parasites inhabit the intestines of dogs and cats (definitive hosts), which excrete eggs in their feces.^2^ When these eggs hatch in freshwater, coracidia are released and subsequently ingested by microscopic crustaceans of the genus *Cyclops* (first intermediate host), leading to the formation of procercariae larvae. Fish, snakes, and amphibians (second intermediate hosts) ingest *Cyclops*, resulting in the development of plerocercoid larvae, known as spargana.^3^ When dogs and cats feed on these intermediate hosts, spargana develop into adult worms that inhabit the intestine. Human infection occurs mainly through ingestion of raw meat from fish or amphibians or by drinking water contaminated with *Cyclops*.^2^ Sparganosis is rare in Brazil, with only a few cases previously reported.^4^ Herein, we report two autochthonous cases.

## Case 1

**Fig. 1:**
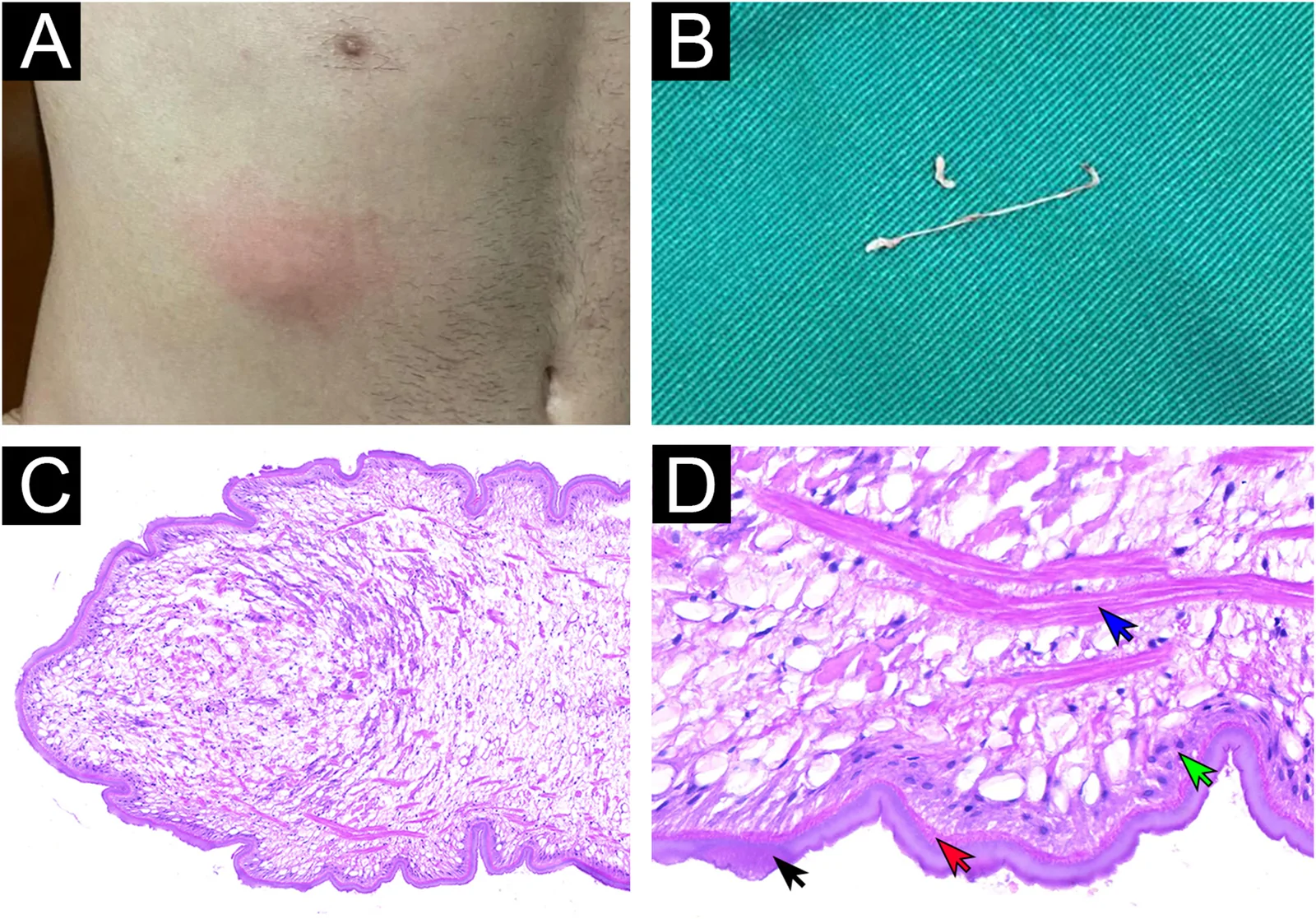
**Sparganosis.** (A) Erythematous subcutaneous nodule measuring approximately 3 cm on the right flank. (B) Macroscopic appearance of a plerocercoid larva of a cestode of the genus Spirometra, showing an elongated, flattened white worm. (C–D) Histopathology of the sparganum. The body wall is composed of tegument (black arrow), two thin layers of muscle cells (red arrow), and tegumentary cells (green arrow). The central portion consists of loose stroma containing muscle fibers (blue arrow) and calcareous bodies (not visible in these images), without identifiable internal organs. (Hematoxylin & eosin, 100× and 400×).

A 34-year-old man from Gurupi, Tocantins, Brazil, presented in 2022 with a firm, indurated, erythematous subcutaneous nodule measuring approximately 3 cm on the right flank, accompanied by smaller nodules covered by normal skin that had appeared 15-days before consultation. He reported pruritus and a sensation of warmth in the lesions and denied fever. Thirty days before lesion onset, the patient had traveled to Ilha do Bananal, a fluvial island in the state of Tocantins, characterized by a hot tropical climate with rainy summers and dry winters, where he consumed raw freshwater fish from Lago Preto (Black Lake; 11°54′33.2″S, 50°38′28.4″W). Abdominal ultrasound suggested an inflammatory-infectious process with thickening of the skin and abdominal wall. Complete surgical excision was performed, with removal of a parasite measuring approximately 4 cm in length. Histopathologic examination demonstrated lobular panniculitis with a mixed inflammatory infiltrate, including eosinophils surrounding an empty tract lacking epithelial lining. A larval structure was identified, externally delimited by a body wall composed of tegument (a homogeneous structure measuring approximately 5–15 μm in thickness), two thin layers of muscle cells, and tegumentary cells. The central portion consisted of loose stroma containing calcareous bodies and muscle fibers, without identifiable internal organs.^5^, ^6^, ^7^ (Fig. 1). These findings allowed identification of the parasite as *Spirometra* sp. Surgical excision was curative.

## Case 2

**Fig. 2:**
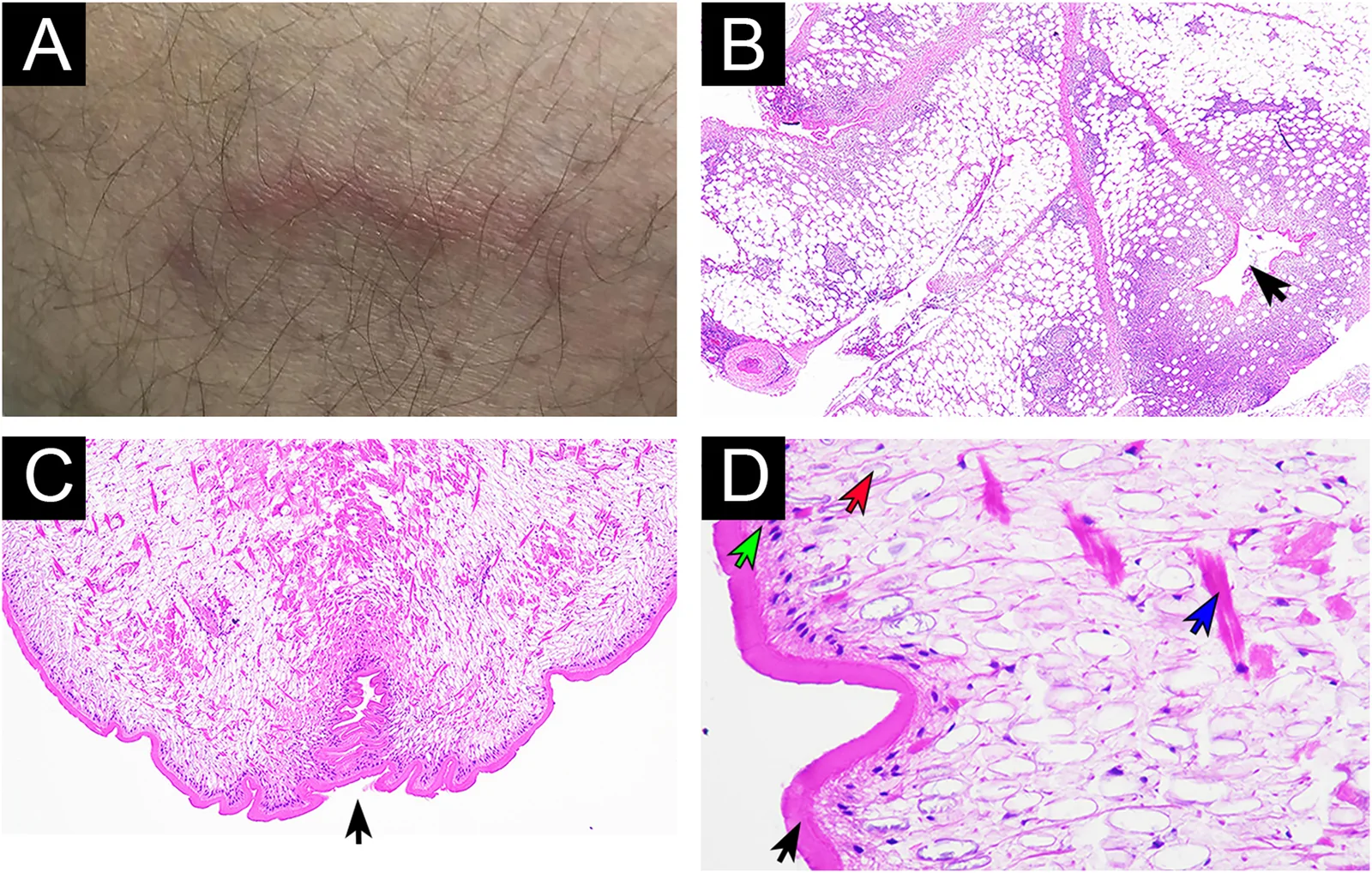
**Sparganosis.** (A) Erythematous serpiginous lesion on the medial aspect of the right thigh. (B) Histopathology revealing lobular panniculitis with a dense mixed inflammatory infiltrate surrounding a tract (black arrow) lacking epithelial lining (Hematoxylin & eosin, 20×). (C) Histologic section of the anterior portion of the worm showing the bothrium (black arrow) (Hematoxylin & eosin, 100×). (D) High-power view of the sparganum. The tegument (black arrow) and tegumentary cells (green arrow) are observed in the body wall, while calcareous bodies (red arrow) and muscle fibers (blue arrow) are present in the central portion of the worm. Note the absence of internal organs (Hematoxylin & eosin, 400×).

A 59-year-old man from Volta Redonda, Rio de Janeiro, Brazil, developed in October 2018 an erythematous serpiginous lesion on the right thigh a few weeks after consuming raw freshwater fish from the Uatumã River, at a site near São Sebastião do Uatumã, Amazonas, Brazil (2°26′23.7″S, 58°14′39.9″W), an area with a hot, humid equatorial climate, abundant year-round rainfall, and only a short dry season. The lesion had an intermittent course with migratory recurrences. Initial laboratory tests revealed mild eosinophilia (7%), which regressed in subsequent years. Multiple treatment cycles with albendazole (800 mg/day for 21-days) were administered following the recommended regimen for gnathostomiasis. These interventions resulted in temporary improvement followed by recurrence. In August 2022, soft tissue ultrasound revealed a tortuous tract in the hypodermis containing a mobile structure. Surgical excision was performed, removing a parasite measuring approximately 5 cm in length. Histopathologic examination revealed a dense mixed inflammatory infiltrate with eosinophils in the hypodermis surrounding an empty tract lacking an epithelial lining. An associated larval structure with typical features of *Spirometra* sp. was identified.^5^, ^6^, ^7^ (Fig. 2). Surgical excision was curative.

Cutaneous sparganosis usually manifests as subcutaneous nodules that may be asymptomatic or tender and occasionally migratory.^8^ Erythematous serpiginous lesions may also occur.^9^ Extracutaneous involvement is less common and results from tissue migration or, rarely, hematogenous dissemination.^8^ Ocular and neurological forms are the most clinically significant because of their associated morbidity and mortality.^8^, ^10^ Other reported sites include the lungs, pleura, abdominal cavity, and urogenital tract.^10^ In Case 2, nearly 4-years elapsed between symptom onset and curative surgical excision. The patient emphatically denied any further exposure after the onset of the first lesion in 2018, including ingestion of untreated water or raw freshwater fish. This prolonged course is biologically plausible, since spargana may remain viable in the human body for up to 20- to 30-years.^3^, ^7^, ^9^, ^11^ The differential diagnosis includes panniculitis, lipomas, cysts, and abscesses, as well as other parasitic infections, such as gnathostomiasis, which has also been reported as autochthonous in Brazil.^12^ Sparganosis and gnathostomiasis share overlapping epidemiologic features related to freshwater exposure and ingestion of raw or undercooked intermediate hosts. Clinically, both may present with migratory subcutaneous lesions, although this pattern is more typical of gnathostomiasis, whereas sparganosis more often presents as a fixed nodule. Histopathologically, *Gnathostoma* is a smaller nematode with identifiable internal organs, whereas sparganum is a larger cestode larva lacking an intestinal tract and containing calcareous bodies.^5^, ^6^, ^7^, ^13^ A history of consuming raw freshwater fish is essential for clinical suspicion, given the strong association between dietary habits and infection risk.^1^, ^8^ The clinical variability and rarity of the disease contribute to underdiagnosis, particularly in non-endemic regions, highlighting the importance of maintaining a high index of suspicion in patients with a compatible epidemiological history.^1^, ^8^ Diagnosis is initially established through macroscopic and microscopic morphological identification of the parasite at the genus level.^2^, ^7^ However, definitive species-level identification requires molecular methods.^14^ Surgical excision is the treatment of choice and is both effective and definitive, as antihelminthic therapy has shown limited efficacy.^1^

These cases aim to increase awareness among dermatologists and dermatopathologists about sparganosis, a rare and possibly underdiagnosed zoonosis in Brazil. The present report highlights its clinical presentation, histopathological findings, parasitological identification, epidemiological context, and therapeutic management.

## ORCID ID

Ana Carolina Cortes Ferreira: 0009-0005-5784-3476

Emyle Vitoria Pereira Sousa: 0009-0001-2996-8168

Mariana Serdeira Arbex Pinto: 0000-0001-9217-0100

Kátia Maria Serdeira Arbex: 0009-0003-8978-7283

## Financial support

None declared.

## Authors’ contributions

Ana Carolina Cortes Ferreira: Intellectual participation in propaedeutic and/or therapeutic management of studied case 1; study conception and planning; manuscript critical review.

Mariana Bezerra Benevides Brandão: Data collection, analysis and interpretation; preparation and writing of the manuscript; study conception and planning.

Emyle Vitória Pereira Sousa: Data collection, analysis and interpretation.

Mariana Serdeira Arbex Pinto: Intellectual participation in propaedeutic and/or therapeutic management of the studied case 2.

Kátia Maria Serdeira Arbex: Intellectual participation in propaedeutic and/or therapeutic management of the studied case 2.

Thiago Jeunon de Sousa Vargas: Intellectual participation in propaedeutic and/or therapeutic management of studied cases 1 and 2; preparation and writing of the manuscript; manuscript critical review; approval of the final version of the manuscript.

## Research data availability

Does not apply.

## Conflict of Interest

None declared.
